# Spinal sagittal imbalance in patients with lumbar disc herniation: its spinopelvic characteristics, strength changes of the spinal musculature and natural history after lumbar discectomy

**DOI:** 10.1186/s12891-016-1164-y

**Published:** 2016-07-22

**Authors:** Chen Liang, Jianmin Sun, Xingang Cui, Zhensong Jiang, Wen Zhang, Tao Li

**Affiliations:** Department of Spine Surgery, Shandong Provincial Hospital Affiliated to Shandong University, No.324 Jing Wu Road, Jinan, 250021 People’s Republic of China

**Keywords:** Spinal sagittal imbalance, Lumbar disc herniation, Spinal musculature, Electromyography

## Abstract

**Background:**

Spinal sagittal imbalance is a widely acknowledged problem, but there is insufficient knowledge regarding its occurrence. In some patients with lumbar disc herniation (LDH), their symptom is similar to spinal sagittal imbalance. The aim of this study is to illustrate the spinopelvic sagittal characteristics and identity the role of spinal musculature in the mechanism of sagittal imbalance in patients with LDH.

**Methods:**

Twenty-five adults with spinal sagittal imbalance who initially came to our clinic for treatment of LDH, followed by posterior discectomy were reviewed. The horizontal distance between C7 plumb line-sagittal vertical axis (C7PL-SVA) greater than 5 cm anteriorly with forward bending posture is considered as spinal sagittal imbalance. Radiographic parameters including thoracic kyphotic angle (TK), lumbar lordotic angle (LL), pelvic tilting angle (PT), sacral slope angle (SS) and an electromyography(EMG) index ‘the largest recruitment order’ were recorded and compared.

**Results:**

All patients restored coronal and sagittal balance immediately after lumbar discectomy. The mean C7PL-SVA and trunk shift value decreased from (11.6 ± 6.6 cm, and 2.9 ± 6.1 cm) preoperatively to (−0.5 ± 2.6 cm and 0.2 ± 0.5 cm) postoperatively, while preoperative LL and SS increased from (25.3° ± 14.0° and 25.6° ± 9.5°) to (42.4° ± 10.2° and 30.4° ± 8.7°) after surgery (*P* < 0.05). The preoperative mean TK and PT (24.7° ± 11.3° and 20.7° ± 7.8°) decreased to (22.0° ± 9.8° and 15.8 ± 5.5°) postoperatively (*P* < 0.05). The largest recruitment order on the level of T7-T8, T12-L1 and the herniated level all improved compared with before and after surgery (*P* < 0.05). All patients have been followed up for more than 2 years. The mean ODI was 77.8 % before surgery to 4.2 % at the final follow-up.

**Conclusions:**

Spinal sagittal imbalance caused by LDH is one type of compensatory sagittal imbalance. Compensatory mechanism of spinal sagittal imbalance mainly includes a loss of lumbar lordosis, an increase of thoracic kyphosis and pelvis tilt. Spinal musculature plays an important role in spinal sagittal imbalance in patients with LDH.

## Background

Sagittal balance is a state in which an individual is capable of keeping a stable standing position with minimal muscle expenditure. It needs several factors interact with each other including bone morphology (spine and pelvic), disc and ligaments mechanical behavior, muscle strength and resistance, and ability of compensation. When one of the factors disrupted, sagittal imbalance occurs [1]. Numerous studies focus on the radiologic parameters to evaluate the state of spinal sagittal imbalance, but it’s blank of the clinical symptoms [2–6]. Lee described the typical symptoms as follows: stooping with walking difficulty, inability to lift heavy objects in front, difficulty in climbing slopes, and the need to support oneself with the elbow when working in the kitchen, resulting in formation of a hard corn on the extensor surface of the elbow [7].

Lumbar disc herniation (LDH) is a common disease, mainly causing low back pain and radiculopathy. But in some patients, the complaint is unable to stand erect, stooping with walking difficulty as well as inability to lift heavy objects in front. The clinical symptom is similar to spinal sagittal imbalance.

However, the etiology, classification, mechanism and radiographic parameters of spinal sagittal imbalance caused by LDH have not been clearly investigated. This study retrospectively reviewed a group of patients with LDH who initially presented sagittal imbalance posture, aimed to investigate their features of sagittal plane, the role of spinal musculature and the effect of posterior discectomy on sagittal imbalance. It will be helpful to understand the main cause of adult degenerative lumbar scoliosis with stenosis.

## Methods

Totally 577 LDH patients were enrolled in our hospital, 25 patients initially presented spinal sagittal imbalance between January 2010 and May 2012. There were 17 males and 8 females with a mean age of 37.4 years (range, 25–55years). Since there is no established criterion to define sagittal imbalance, we decided to combine the symptom with a radiographic parameter. The patients all stood and walked in a forward bending posture. We use the horizontal distance between C7 plumb line and the posterior superior corner of S1 (SVA) as the radiographic standard. The horizontal distance between C7PL and S1 SVA greater than 5 cm anteriorly or posteriorly are considered as sagittal imbalance [8]. In this study, we define the distance greater than 5 cm anteriorly as sagittal imbalance. We excluded patients with neuromuscular diseases; ankylosing spondylitis; flat-back syndrome; history or clinical signs of hip, pelvic or lower limbs;previous spine surgery; spinal compression fractures, metabolic bone disease, infection or tumor. More than one level lumbar disc herniation is also excluded.

Lumbar disc herniation was confirmed by means of computed tomography (CT) or magnetic resonance imaging (MRI). The disc herniation located at L1-2 in 1 patient, L3-4 in 6 patients, L4-L5 in 13 patients, L5-S1 in 5 patients.

In all patients, traditional conservative measures had failed, including bed rest, physical therapy, use of nonsteroidal medications, and modification of lifestyle prior to surgery for more than 3 months.

Radiographic evaluation mainly included standard 36-inch digital standing lateral and anterior–posterior radiographs of the entire spine and pelvis obtained one day before surgery, at the immediate postoperative period (one day after operation), 3 months after surgery and at the latest clinical follow-up. Standing AP radiographs were obtained with the knees and hips fully extended and hands resting at waist height. Standing lateral radiographs were taken with fingers on the clavicles and shoulders in 45° of forward elevation, and knees and hips fully extended [8–10]. Before surgery, as some patients can stand erect for a few seconds, we asked these patients to stand or walk until they were unable to stand erect and took the radiographs exactly at that moment.

All the patients received surgeries performed by three experienced surgeons in our department. Selective discectomy were performed through transforaminal percutaneous approach and under direct endoscopic visualization. The operation was performed with the patient in the prone position and under local anesthesia. Estimated blood loss: < 20 ml, operation time:45–75 min (62.88 min), hospital stay: 8-12d (8.64d). No complications were observed.

### Radiographic measurements

Parameters to evaluate the balance in the sagittal alignment are as follows: for the pelvis: pelvis incidence (PI), pelvis tilt (PT), sacral slope (SS); for the spine: sagittal vertical axis (SVA), thoracic kyphosis (TK), lumbar lordosis (LL) [11]. Trunk shift (TS) is valued as the only parameter to access the balance in the coronal alignment. Table 1 shows the abbreviations and descriptions of the radiographic parameters.

**Table 1:** Parameters of sagittal spino-pelvic alignment

Parameter	Abbreviation	Descriptions
C7 plumb line- sagittal vertical axis (cm)	C7PL-SVA	Horizontal distance between the posterior corner of the sacrum and the C7 plumb line^a^
Trunk shift (cm)	Trunk shift	Horizontal distance between the C7 plumb line and the Center Sacral Vertical Line^b^
Thoracic kyphosis (°)	TK	Cobb angle method between T5 and T12
Lumbar lordosis (°)	LL	Cobb angle method between L1 and S1
Pelvic incidence (°)	PI	Angle between the perpendicular line from the sacral plate and the line connecting the midpoint of the sacral plate to the bicoxofemoral axis
Pelvic tilt (°)	PT	Angle between the line connecting the midpoint of the sacral plate to the bicoxofemoral axis and the vertical plane
Sacral slope (°)	SS	slope angle between superior endplate of S1 and horizontal line

^a^Value is considered negative when the C7 plumb line is posterior to the posterior corner of the sacrum

^b^Value is considered negative when the C7 plumb line is left to the Center sacral vertical line

### Measurement of back muscle strength

Back muscle strength from the maximal voluntary contraction of the paraspinal muscles is determined by the use of electromyography(EMG). Excessive body hair was shaved, and the skin was cleaned with water and alcohol swabs before electrode positioning. NIHON KOHDEN 9200 electromyography instrument was used in this test. Disposable surface adhesive electrodes were placed on both sides of the spine. Three different levels represent the thoracic, thoracolumbar, and herniated disc level respectively. The average value was calculated and recorded.

The EMG signals from the back muscle are collected (filter 20Hz-10kHz, scanning rate 0.1/Div, sensivity 0.1mv/Div) with a pair of Ag/AgCl bipolar standard surface adhesive electrodes. The electrodes were positioned 2 cm lateral to the spinous process at three different levels (T7/8, T12/L1 and the herniated level, positioned according to the anatomic signs). A ground reference electrode was attached to the ulnar styloid process [12]. The patients lay prostrate on the bed in a relaxed situation. We asked them to stretch to the most with the paraspinal muscles thrice in each level. The mixed recruitment potentials were recorded, and the mean largest recruitment potential was calculated and recorded. There was a one-minute interval to relax the back muscles before switching to the next level.

After surgery, all the patients were kept lying on horizontal firm bed and then gradually mobilized and straight-leg raising until they were ambulatory (1–2 days after operation). We instructed all patients to rehabilitate in our ward and discharged them 7–10 days after surgery. A small suitable lumbosacral corset was essential for them when they began to walk. None of the patients underwent waist musculi dorsi function exercise until the post-op EMG measurement. The Chinese version of the Oswestry Disability Index (ODI) was available, in which Section 8 (sex life) was omitted. We expressed the total score as a percentage, thereof 0 % represents no pain and disability and 100 % equals the worst pain and disability [13].

### Statistical analysis

SPSS 22. 2 package software was used for all statistical analysis. *P* values were derived from Student’s paired *t* test comparison of preoperative and postoperative measurements. *P* values less than 0.05 were considered statistically different.

## Results

All the follow-up data is obtained. The final follow-up time in these patients varied between 24 months and 38 months after surgery (mean, 28.6 months). No patients needed pain medication at follow-up. Before surgery, the mean ODI was 77.8 % (range, 66.7–88.9 %), while at the final follow-up, it improved to 4.2 % (range, 0–13.3 %). Detailed preoperative and postoperative (1 day post-operation) radiographic results were shown in Table 2.

**Table 2:** Demographics and preoperative and postoperative radiographic parameters of patients

No	Age	Sex	Level	C7PL-cm		TK°		LL°		SS°		PT°		PI°	TS-cm	
				Pre	PO	Pre	PO	Pre	PO	Pre	PO	Pre	PO		Pre	PO
1	36	F	4/5	7.8	−4.3	24	16	28	40	23	27	31	27	54	1.6	0.6
2	33	M	4/5	18.3	−0.5	9	13	7	30	19	30	21	10	40	−0.4	0
3	41	M	3/4	5.3	−1.1	46	35	51	46	40	30	4	14	44	3.5	0.2
4	33	F	5/1	2.7	−5.1	24	29	20	27	7	12	12	7	19	7.9	0.4
5	53	M	3/4	13.8	0.5	37	40	30	64	32	44	28	16	60	8.1	0.7
6	45	M	4/5	11.2	0.2	29	21	38	50	35	39	20	16	55	11.5	0.4
7	35	M	1/2	22.3	0.5	22	26	21	49	30	40	28	18	58	1.9	0
8	55	M	3/4	23.6	2.4	15	10	9	43	23	30	22	15	45	6.2	0
9	32	F	5/1	7.5	−3.9	25	17	30	40	21	26	32	27	53	1.8	0.7
10	35	M	4/5	18	−0.6	10	15	7	32	18	29	20	9	38	0.5	0
11	39	M	4/5	5.9	−0.6	45	34	49	45	38	29	5	14	43	3.0	0.2
12	23	F	5/1	3	−4.8	21	29	16	25	9	15	12	6	21	6.9	0.9
13	44	M	4/5	22.9	2.5	16	12	10	44	25	32	22	15	47	6.4	0.1
14	41	M	3/4	9	−2.1	27	20	36	49	34	39	23	18	57	−8.2	0
15	36	M	5/1	12	0.5	31	24	39	52	36	40	21	17	57	12.0	0.5
16	45	M	5/1	19.7	2.5	15	11	8	42	26	32	21	15	47	−6.0	0
17	25	F	4/5	6.9	−3.9	23	16	28	39	20	31	31	20	51	1.2	0.2
18	48	M	4/5	14	0.5	36	39	30	63	35	43	25	17	60	−6.9	−0.3
19	31	M	3/4	6.2	−0.4	49	38	52	46	39	30	9	18	48	3.8	0.2
20	26	F	4/5	3.2	−5.0	25	30	19	27	10	12	11	9	21	6.9	0.2
21	27	M	4/5	10.9	2.4	6	9	12	30	21	26	21	16	42	7.9	1.0
22	49	F	4/5	9.9	2	17	22	16	45	24	23	25	26	49	−10.5	−1.5
23	25	F	4/5	6	1.5	20	15	26	36	20	27	26	19	46	−2.5	−0.6
24	36	M	4/5	10.2	0.9	29	20	38	48	34	41	22	15	56	11.1	0.4
25	42	M	3/4	19.8	2.2	17	10	12	48	20	33	25	12	45	5.6	0

For the entire spine, there was a significant difference in SVA preoperatively and postoperatively with the value decreased from (11.6 ± 6.6 cm) to (2.9 ± 6.1 cm) (*P* < 0.05). The average of trunk shift distance was (2.9 ± 6.1 cm) and (0.2 ± 0.5 cm) before and after surgery, which was significantly different (*P* < 0.05). Thoracic kyphotic angle decreased significantly from (24.7° ± 11.3°) to (22.0° ± 9.8°) comparing before with after surgery (*P* < 0.05). The Lumbar lordotic angle improved significantly from (25.3° ± 14.0°) to (42.4° ± 10.2°) comparing before with after surgery (*P* < 0.05).

In terms of the pelvic parameters, the mean sacral slope angle increased from (25.6° ± 9.5°) before survey to (30.4° ± 8.7°) immediately after survey. And the mean pelvic tilt angle deceased from (20.7° ± 7.8°) to (15.8° ± 5.5°). Similarly, there were significant differences between preoperative and postoperative values in terms of the SS and PT (*P* < 0.05) (Table 3).

**Table 3:** Comparison of the preoperative and postoperative spinopelvic parameters (mean ± standard deviation)

Parameter	Preoperative	Postoperative	*P*
C7PL-SVA(cm)	11.6 ± 6.6	0.5 ± 2.6	<0.05
TK(°)	24.7 ± 11.3	22.0 ± 9.8	<0.05
LL(°)	25.3 ± 14.0	42.4 ± 10.2	<0.05
P I(°)	46.2 ± 11.6	46.2 ± 11.6	–
PT(°)	20.7 ± 7.8	15.8 ± 5.5	<0.05
SS(°)	25.6 ± 9.5	30.4 ± 8.7	<0.05
TS (cm)	2.9 ± 6.1	0.2 ± 0.5	<0.05

**Fig. 1:**
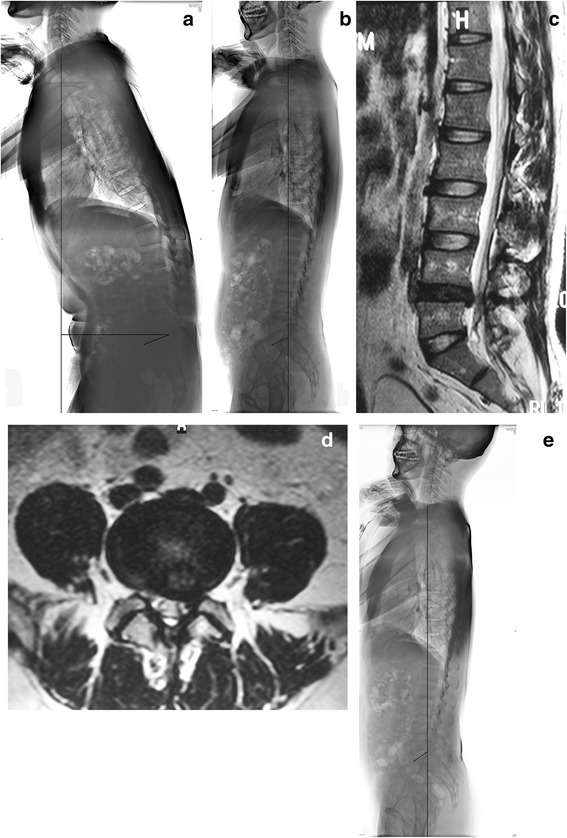
Thirty-three year-old man with L4/5 disc herniation (patient No.2). **a** Preoperative lateral radiograph showed sagittal imbalance. (SVA 18.3 cm). **b** Immediately after surgery, radiograph showed restored sagittal balance. (SVA −0.5 cm). **c**, **d** MRI revealed L4/5 disc herniation with impingement of the left L5 nerve root. **e** Lateral radiograph taken 2.5 years after surgery showed sagittal balance. (SVA 0.5 cm)

**Fig. 2:**
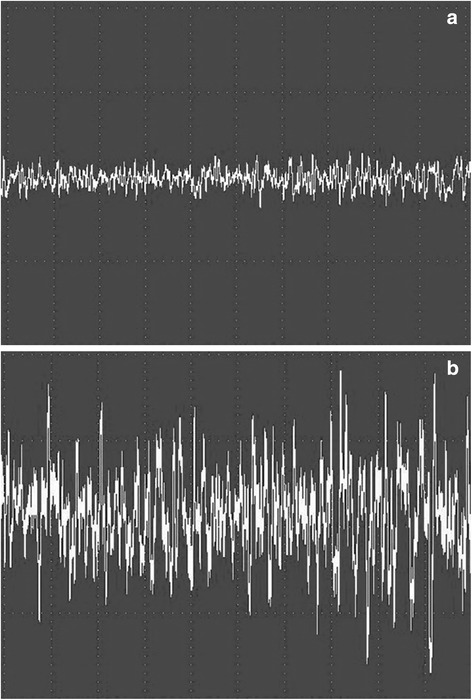
Changes of the largest recruitment order of surface EMG (patient No.2). **a** before surgery. **b** immediately after surgery

Before surgery, mean largest recruitment potential of the spinal musculature in the herniated level is smaller than the other two levels. After surgery, there were significant differences of the largest recruitment potentials of the spinal musculature in all the three levels (Table 4). It is noticed that largest recruitment potentials in the herniated level turned out to be the biggest among the three. Changes of the radiographic and EMG parameters (patient No.2) are shown (Figs. 1 and 2).

**Table 4:** Comparison of the largest recruitment orders of the spinal musculature before and after surgery (mean ± standard deviation)

Largest recruitment order(mv)	T7-T8	T12-L1	L4-L5*
Before surgery	0.43 ± 0.24	0.46 ± 0.21	0.32 ± 0.17
After surgery	0.76 ± 0.34	0.73 ± 0.17	0.95 ± 0.31
*P*	<0.05	<0.05	<0.05

L4-L5*represents the herniated level

## Discussion

There are multiple causes resulting in the sagittal imbalance. Reduced muscle strength, adjacent disc degeneration, and hip and pelvic disease may contribute to a decrease of the patient’s ability to compensate and may result in increased disability [14].

The present study demonstrated that compared to postoperative changes of the parameters, the anterior translation of the C7 plumb line, loss of lumbar lordosis, thoracic kyphosis and sacral slope improved well. Immediately after surgery, evaluating by SVA, every patient restored sagittal balance.

The largest recruitment potential of the spinal musculature after the operation obviously increased in all the three levels. Furthermore, after surgery, all patients were pain-relived and improved life quality significantly. It is obvious to conclude that spinal sagittal imbalance caused by LDH is influenced by decreased trunk muscle strength (especially extensor strength). Since all the operations were done through the transforaminal percutaneous approach, the trunk muscle per se is not changeable before and after surgery. In patients with LDH, the weakness of muscles is likely to result from back pain and radiculopathy. Once the pain is relieved, the strength of muscles improves. Back pain and sciatic pain limit the activity of the muscle thus leading to the weakness of the muscle and consequently the spinal sagittal imbalance. This once more indicted that the sagittal imbalance is not a structural imbalance, but a compensatory one.

A good understanding of the mechanisms is helpful for surgeons to optimize the management when making surgical plans. Some patients with sagittal imbalance in the x-rays may have no symptom for a long time but present the imbalance symptom suddenly sometimes. This kind of patients may have severe degenerative lumbar diseases. Lumbosacral nerve root compression syndrome contributes to the development of sagittal imbalance. Our study is helpful to understand this complicated condition. For example, a patient with degenerative lumbar scoliosis may maintain a balanced sagittal alignment with strong support of trunk muscle. However, once he got lumbar disc herniation, he would have performed obvious sagittal imbalance. As a result, when making plans to restore his spinal balance, we can do a single posterior discectomy rather than osteotomy.

Authors have evaluated the morphology and orientation of pelvis in asymptomatic adults, obtaining different mean normal values [14–17]. However, sagittal spinal morphology is specific to each patient, influenced by age, sex, weight, and ethnicity, thus differing from one individual to another. A standard sagittal balance does not exist in the normal population [18, 19]. So the present study chose the same patients before and after surgery as self-control.

After having reviewed all the compensatory mechanisms of sagittal imbalance in the literature, Barrey et.al proposed a three steps algorithm to analyze the balance state and summarized three common compensatory mechanisms. The three main steps algorithm referred to measuring the value of PI, evaluating global sagittal alignment by analyzing the position of C7 and determining the compensatory mechanisms including retrolisthesis, knee flexion and inadequent PT to PI [20]. Results in our study are in good agreement with his theory. Knee flexion has already been reported as an important compensatory mechanism in lower limbs area, so in our study we ask all the patients to fully extend their knees to better express the imbalance state.

It is well-known that different standing positions and arm positions have effects on the measurement of C7 plumb line. Standing lateral radiographs were taken strictly according to the given criteria ‘fingers on the clavicles and shoulders in 45° of forward elevation, and knees and hips fully extended’.

This study is the first attempt to evaluate the spinal sagittal imbalance by the strength of extensor muscles. C. Lamartina and P. Berjano presented a classification of sagittal imbalance based on the level of the deformity and mentioned that inadequate muscle strength is one of the compensatory mechanisms [21]. However, there is no evidence to prove it. Surface-EMG is a noninvasive examination approach to record the electrical activity. Surface-EMG has been proved as a valid and reliable way to assess the neuromuscular response of the pelvic floor muscles and back muscles. It has been widely used in the assessment of back muscle weakness, muscle fiber composition and fatigability [22–25]. The most common index of surface-EMG are IMF (initial median frequency) and IRMS (initial EMG root mean square) [26–28]. The largest recruitment order is a common indicator to evaluate the strength of muscle.

In degenerative flat back, sagittal imbalance was more evident when walking, hitting its dynamic nature. Similarly, through our study, we also found the dynamic nature of sagittal imbalance in patients with LDH. All the patients have the typical symptoms of sagittal imbalance, however in the standing lateral radiographs C7PL sometimes fell behind the posterior superior corner of S1 showing no imbalance. This is because the radiographs only revealed the static status of the spine. As a result, we asked the patients to walk until they were unable to stand erect and took the radiographs exactly at that moment. Those patients only walked a few steps. To some extent, the radiographs could show the dynamic features of sagittal imbalance. This method is similar with the gait analysis to evaluate the dynamic sagittal imbalance [29, 30].

Being in the state of spinal sagittal imbalance for a long time may lead to the degenerative regression and hyperosteogeny in the spine and atrophy of the paraspinal muscle. Abnormal postures resulted from this vicious cycle may convert previous compensative spinal sagittal imbalance into a structural one. At that time, it becomes much more difficult to treat.

In addition, limitations of this study lie in the inherent errors in measuring the radiographic parameters and EMG values. Further study is needed to focus on mechanism turning the compensative sagittal imbalance into the structural one. More surface-EMG parameters should be used to assess the changes of the muscle before and after surgery.

## Conclusion

Spinal sagittal imbalance caused by LDH is one type of compensatory sagittal imbalance. Compensatory mechanism of spine sagittal imbalance mainly includes a loss of lumbar lordosis, an increase of thoracic kyphosis and pelvis tilt. Trunk muscle plays an important role in maintaining the spinal coronal and sagittal balance. Early posterior discectomy can provide a great opportunity for spontaneous correction of sagittal imbalance.

## Abbreviations

C7PL-SVA, C7 plumb line-sagittal vertical axis; CT, computed tomography; EMG, electromyography; HRQOL, health related quality of life; IMF, initial median frequency; IRMS, initial EMG root mean square; LDH, lumbar disc herniation; LL, lumbar lordosis; MRI, magnetic resonance imaging; ODI, oswestry disability index; PI, pelvis incidence; PT, pelvis tilt; SS, sacral slope; SVA, sagittal vertical axis; TK, thoracic kyphosis; TS, trunk shift
